# The endocannabinoid system: ‘NO’ longer anonymous in the control of nitrergic signalling?

**DOI:** 10.1093/jmcb/mjx008

**Published:** 2017-01-24

**Authors:** Christopher Lipina, Harinder S. Hundal

**Affiliations:** 1 Division of Cell Signalling and Immunology, Sir James Black Centre, School of Life Sciences, University of Dundee, DundeeDD1 5EH, UK

**Keywords:** endocannabinoid system, reactive nitrogen species, nitric oxide, nitrosative stress, cannabinoid receptor

## Abstract

The endocannabinoid system (ECS) is a key cellular signalling system that has been implicated in the regulation of diverse cellular functions. Importantly, growing evidence suggests that the biological actions of the ECS may, in part, be mediated through its ability to regulate the production and/or release of nitric oxide, a ubiquitous bioactive molecule, which functions as a versatile signalling intermediate. Herein, we review and discuss evidence pertaining to ECS-mediated regulation of nitric oxide production, as well as the involvement of reactive nitrogen species in regulating ECS-induced signal transduction by highlighting emerging work supporting nitrergic modulation of ECS function. Importantly, the studies outlined reveal that interactions between the ECS and nitrergic signalling systems can be both stimulatory and inhibitory in nature, depending on cellular context. Moreover, such crosstalk may act to maintain proper cell function, whereas abnormalities in either system can undermine cellular homoeostasis and contribute to various pathologies associated with their dysregulation. Consequently, future studies targeting these signalling systems may provide new insights into the potential role of the ECS**–**nitric oxide signalling axis in disease development and/or lead to the identification of novel therapeutic targets for the treatment of nitrosative stress-related neurological, cardiovascular, and metabolic disorders.

## Introduction

**Figure 1:**
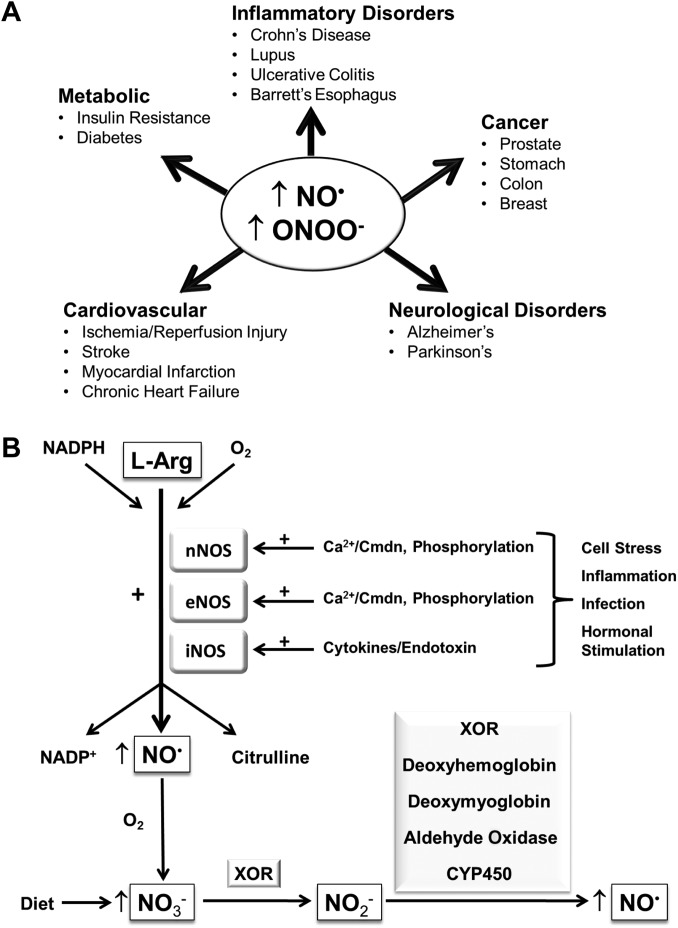
NO^•^ production and its involvement in disease pathogenesis. (**A**) Schematic diagram illustrating the involvement of increased production of NO^•^ and associated RNS (e.g. ONOO^–^) in the development of various pathologies. (**B**) NO^•^ biosynthesis is predominantly catalyzed by three isoforms of NOS that exhibit distinct activation mechanisms and tissue distributions: namely nNOS, iNOS, and eNOS. All three NOS isoforms use L-arginine as a substrate, which is converted into NO^•^ and citrulline, and oxygen and NADPH as co-factors. The enzymes nNOS and eNOS are constitutively expressed in mammalian cells and synthesize NO^•^ in response to elevated intracellular calcium by increasing calmodulin (Cmdn) binding to NOS, although they may also be activated or inhibited through their phosphorylation by upstream protein kinases. In contrast, iNOS protein is either very low or undetectable in most cell types; however, stimulation with, for example, cytokines or endotoxins, can lead to increased iNOS gene transcription, resulting in enhanced production of NO^•^ in certain cell types (e.g. immune cells). Alternatively, NO^•^ can also be generated by the enzyme-mediated reduction of NO_3_^–^ and NO_2_^–^, anions derived from the oxidation of NO^•^ or through dietary sources, as indicated. CYP450, cytochrome P450. The plus sign (+) denotes a stimulatory effect.

Nitric oxide (NO^•^) is a bioactive free radical produced by most cell types, which can serve either as a beneficial physiologic messenger or as a toxic intermediate involved in disease progression (Schmidt and Walter, 1994). Indeed, since its discovery as a key endogenous signalling molecule in mammalian cells, the chemical biology of NO^•^ and its impact upon cellular function has been an important research topic for several decades. It is now widely recognized that many of its biological actions are mediated through its ability to regulate various signalling pathways and/or by altering protein function through post-translational modifications (McDonald and Murad, 1996; Azad et al., 2006; Campanella et al., 2016; Seneviratne et al., 2016). As a consequence of these biological actions, elevated or reduced NO^•^ bioavailability has been implicated in the aetiology of a number of pathological events including the development of various neurodegenerative, metabolic, cardiovascular, and inflammatory disorders (Figure 1A) (Heales et al., 1999; Duncan and Heales, 2005; Pacher et al., 2007). Consequently, there is growing interest in identifying cellular pathways and/or processes that can regulate the levels of NO^•^ and other derived reactive nitrogen species (RNS). Herein, we discuss evidence that supports a role for the endocannabinoid system (ECS), whose activity is determined by endogenous bioactive lipids, in the regulation of nitrergic signalling and highlight emerging evidence pertaining to the involvement of RNS in the modulation of ECS function.

## NO^•^ synthesis in biological systems

The endogenous production of NO^•^ is mainly driven by the two-step oxidation of L-arginine, which can be catalyzed by one of three isoforms of nitric oxide synthase (NOS), namely the neuronal (nNOS), endothelial (eNOS), and inducible (iNOS) isotypes (Knowles et al., 1989; Boucher et al., 1999; Alderton et al., 2001) (Figure 1B). Notably, nNOS and eNOS are constitutive enzymes that require calcium and calmodulin for their activation (Busse and Mulsch, 1990; Abu-Soud et al., 1994; Salerno et al., 1997; Forstermann and Sessa, 2012). In contrast, iNOS activation largely involves its transcriptional upregulation in response to pro-inflammatory stimuli (Figure 1B), which may be important in situations where the rapid production of a large flux of NO^•^ is necessary, for example in stimulated immune cells (Xie et al., 1994; Forstermann and Sessa, 2012). Furthermore, phosphorylation constitutes an additional mechanism for regulating NOS activity, whereby the catalytic activity of the nNOS enzyme has been shown to decrease following its phosphorylation by cyclic adenosine monophosphate (cAMP)-dependent protein kinase (Brune and Lapetina, 1991; Bredt et al., 1992), protein kinase C (Nakane et al., 1991; Bredt et al., 1992), or Ca^2+^/calmodulin-dependent protein kinase II (CAMKII) (Nakane et al., 1991; Bredt et al., 1992; Komeima et al., 2000).

It is important to highlight that NO^•^ can also be produced via the reduction of the inorganic nitrate (NO_3_^–^) and nitrite (NO_2_^–^) anions, which assimilate as end-products of the classic L-arginine**–**NOS pathway following rapid oxidation of NO^•^ and are consumed through dietary sources (Figure 1B). Indeed, several mammalian enzymes have been reported to reduce inorganic nitrate and/or nitrite leading to the generation of NO^•^, including xanthine oxidoreductase (XOR), mitochondrial aldehyde oxidase, and cytochrome P450 (Godber et al., 2000; Kozlov et al., 2003; Li et al., 2008). In addition, nitrite reductase activities have also been reported to be exhibited by deoxyhemoglobin in blood and deoxymyoglobin in cardiac muscle (Cosby et al., 2003; Shiva et al., 2007). Therefore, such enzymes may act to ensure an adequate supply of bioactive NO^•^ under conditions, where the activity of NOS enzymes becomes impaired.

## NO^•^ and its impact on protein and cellular function

There is growing recognition that NO^•^ and other nitrogen-containing free radicals derived from it can act as key signalling effectors. For example, NO^•^ has been identified as a ligand of the soluble guanylyl cyclase, which in turn stimulates the production of cyclic guanosine monophosphate, a key second messenger implicated in the activation of protein kinase G (McDonald and Murad, 1996). Moreover, NO^•^ can also alter the activity of proteins by promoting various post-translational modifications. Firstly, NO^•^ has been shown to induce S-nitrosylation of cysteine thiol groups (in the presence of an electron acceptor to form S–NO bonds) in a variety of proteins including Parkin, Bcl-2, and caspase-3, thereby leading to changes in cell growth capacity, mitochondrial function, and cell survival (Mannick et al., 2001; Azad et al., 2006; Sunico et al., 2013). Indeed, different mechanisms of nitrosylation have been described, including *trans*-nitrosylation by small molecular weight NO^•^ carriers, such as S-nitrosoglutathione, as well as metalloprotein-catalyzed S-nitrosylation (Gaston et al., 2003; Doulias et al., 2010; Evangelista et al., 2013). Conversely, S-nitrothiols can be degraded by various enzymes including the S-nitroglutathione reductase and thioredoxin systems, which can confer protection against nitrosative stress (Benhar et al., 2009). It is important to highlight the distinction between nitrosylation, which is defined as the direct addition of NO^•^ to a reactant, and nitrosation, which refers to the addition of a nitrosonium ion (NO^+^) to a nucleophilic group, such as a thiolate. There is increasing evidence that now supports protein nitrosation as an important mechanism in the regulation of cellular function and disease pathology, including the development of cardiovascular and neurodegenerative disorders (Soetkamp et al., 2014; Seneviratne et al., 2016). In addition to S-nitrosylation and nitrosation, the process of nitration, i.e. the incorporation of a nitro group (–NO_2_) into amino acid residues, has similarly been reported to convey marked structural and/or functional alterations in proteins such as the molecular chaperones heat shock protein 60 (HSP60) and HSP90 (Franco et al., 2015; Campanella et al., 2016). Notably, further NO^•^-mediated alterations to protein function can occur through the formation of derived RNS such as peroxynitrite (ONOO^–^), a highly reactive anion generated by the reaction of NO^•^ with the superoxide anion. Indeed, the accumulation of peroxynitrite has been shown to promote post-translational modifications including protein nitration or thiol oxidation (Quijano et al., 1997) and perturb cellular function by inducing lipid peroxidation (Radi et al., 1991) or through its damaging effects on DNA and mitochondrial integrity (Radi et al., 2002; Radovits et al., 2007). Therefore, signalling mediated through NO^•^ has been shown to impact on protein and cellular function in a number of different ways.

## The ECS

**Figure 2:**
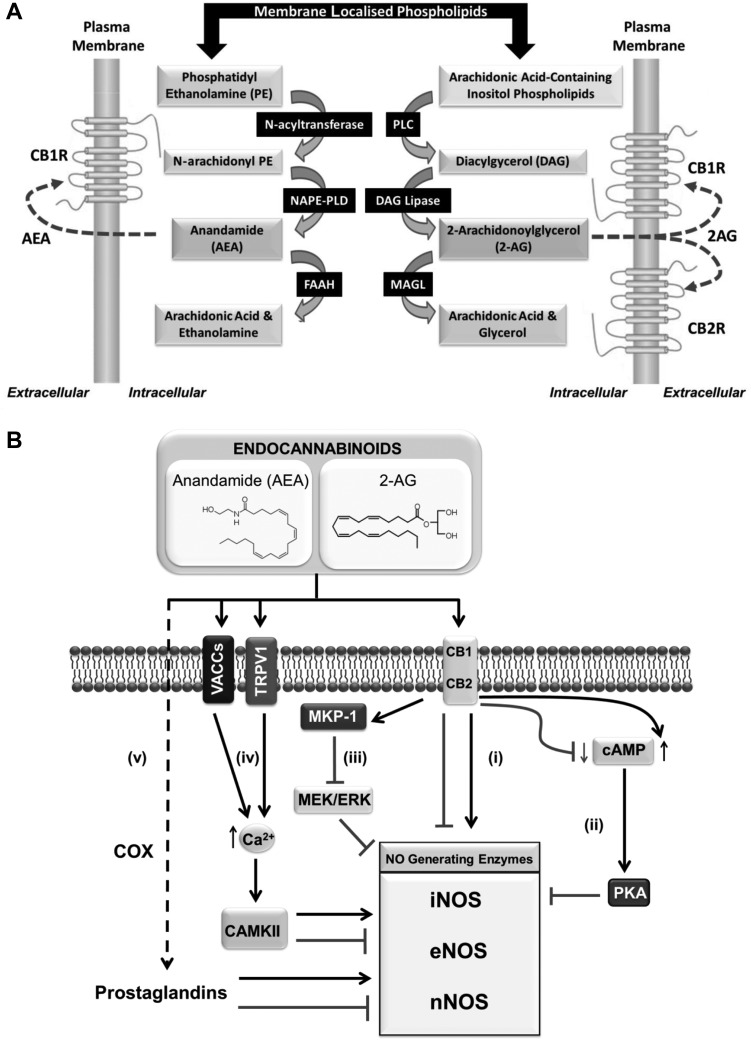
Major pathways involved in the biosynthesis of endocannabinoids and the modulation of NO^•^ production by the ECS. (**A**) Schematic showing the principal enzymes implicated in the synthesis of the endocannabinoids AEA and 2-AG, which in turn can act on target receptors such as CB1R and/or CB2R in order to convey their biological actions. (**B**) Activation of CB1R and/or CB2R has been reported to either stimulate or repress the activity of enzymes implicated in NO^•^ synthesis as indicated (i). Cannabinoid receptor activation can lead to either the activation or impairment of intracellular cAMP levels depending on cell type, which in turn may accentuate or relieve PKA-mediated inhibition of NOS-generating enzymes, respectively (ii). Alternatively, CB1R stimulation has been reported to repress NOS activity through activating the protein phosphatase MKP-1 (iii). Endocannabinoids such as AEA have also been shown to target the vanilloid receptor TRPV1 and voltage-activated calcium channels (VACCs) (iv). In these cases, TRPV1/VACC activation would promote elevations in intracellular calcium signalling that may subsequently impact upon the activity of NOS enzymes, for example by regulating CAMKII activity and/or calmodulin binding. Following their uptake into cells, endocannabinoids can also be further processed (by the action of COX enzymes) into derived metabolites such as prostaglandins (eicosanoids), which have also been shown to modulate NOS activity (v).

The ECS is a ubiquitous ligand-directed signalling system, which has been implicated in regulating a wide range of physiological processes and pathologies, including energy homoeostasis, cardiovascular disease, cancer, and neurodegeneration (Di Marzo and Petrocellis, 2006; Mukhopadhyay et al., 2010b; Oddi et al., 2012). Two key lipid-derived molecules that act as endogenous ligands for this system are anandamide (N-arachidonoylethanolamine, AEA) and 2-arachidonoylglycerol (2-AG)—commonly referred to as endocannabinoids. Both AEA and 2-AG can be synthesized on demand within the plasma membrane from arachidonic acid-derived lipids (Basavarajappa, 2007; Alger and Kim, 2011) (Figure 2A). AEA generation from its membrane phospholipid precursor N-acylphosphatidylethanolamine (NAPE) is driven by the action of the enzyme NAPE-hydrolyzing phospholipase D (NAPE-PLD) (Okamoto et al., 2004). In contrast, phospholipase C (PLC)-mediated cleavage of membrane phosphatidylinositols gives rise to a diacylglycerol (DAG) precursor whose subsequent hydrolysis (via DAG lipase activity) permits the formation of 2-AG (Ueda et al., 2011). In addition to these synthetic pathways, enzymes that catalyze the degradation of AEA and 2-AG have also been characterized, including fatty acid amide hydroxylase (FAAH) and monoacylglycerol lipase (MAGL), respectively (Taschler et al., 2011). Furthermore, several oxidative enzymes, including lipoxygenases, cytochrome P450 monooxygenases, and cyclooxygenase-2 (COX-2), can metabolize endocannabinoids into bioactive derivatives such as eicosanoids, which may function to mediate some of the biological actions of endocannabinoids (Almada et al., 2015; Urquhart et al., 2015; Zelasko et al., 2015).

Both AEA and 2-AG evoke cellular and physiological responses through binding to and activating two distinct G protein-coupled receptors identified as the cannabinoid type 1 (CB1R) and type 2 (CB2R) receptors (Matsuda et al., 1990; Munro et al., 1993). Various synthetic CB1R and/or CB2R agonists (e.g. CP 55,940, ACEA, WIN 55,212-2, JWH-133, and HU-210) have been used to provide mechanistic insight into the regulation of energy homoeostasis by the ECS (Table 1) (Mechoulam et al., 1995; South and Huang, 2008; Deveaux et al., 2009; Lipina et al., 2010; O'Hare et al., 2011). Importantly, these are often applied in combination with selective receptor antagonists to determine receptor-specific responses. Such cannabinoid receptor blockers act by competitively binding and preventing activation of a receptor by an agonist (i.e. as an antagonist) and/or function as inverse agonists through supressing spontaneous (ligand-free) receptor signalling. For example, SR141716 (also known as rimonabant) has been shown to act as both CB1R antagonist and inverse agonist (Bouaboula et al., 1995; Landsman et al., 1997). Notably, endocannabinoids have also been reported to mediate some of their biological effects through alternative molecular targets such as the orphan G protein-coupled receptor GPR55, the cation channel transient receptor potential cation channel subfamily V member 1 (TRPV1), and the peroxisome proliferator-activated receptor (PPAR) α and γ isoforms (O'Sullivan, 2007; Miyashita et al., 2012; Sharir et al., 2012).

**Table 1:** Synthetic modulators of cannabinoid receptor function.

Name	Activity at CB1 (Ki in nM)	Activity at CB2 (Ki in nM)	Comments	References
ACEA	1.4 ± 0.3	>2000	Selective CB1 receptor agonist	Lipina et al. (2010), Tedesco et al. (2010)
AM251	7.5	2000**–**3000	Selective CB1 receptor antagonist/inverse agonist	Eckardt et al. (2009)
SR141716	1.8 ± 0.2	**–**	Selective CB1 receptor antagonist/inverse agonist	Jbilo et al. (2005), Huang et al. (2010)
JWH-133	680	3.4	Selective CB2 receptor agonist	Li et al. (2013)
AM-630	5.2 × 10^3^	31.2	Selective CB2 receptor antagonist/ inverse agonist	Deveaux et al. (2009)
CP 55,940	0.5 ± 0.1	2.8 ± 0.4	Non-selective potent CB1/2 receptor agonist	South and Huang (2008)
HU-210	0.1**–**0.7	0.2**–**0.5	Non-selective potent CB1/2 receptor agonist	Athanasiou et al. (2007)
WIN 55,212-2	4.4 ± 1.3	1.2 ± 0.25	Non-selective CB1/2 receptor agonist	Gustafsson et al. (2006)

Citations refer to studies performed using the compounds listed in order to elucidate the functional role of CB1R and/or CB2R.

## Modulation of nitrergic signalling by the ECS

Growing evidence indicates that ECS ligands are able to regulate the formation and/or release of NO^•^ and derived RNS, in an either positive or negative manner depending upon cell type and stimulus, by acting on distinct molecular targets (Figure 2B and Table 2). In the following sections, we discuss the role of these ECS modulators in regulating NO^•^ formation via CB1R, CB2R, and/or alternative molecular targets.

**Table 2:** Effects of ECS modulation upon NO^•^ production in different cell types and tissues.

Cell type/tissue	Receptor/target mediating response	NO^•^ production and/or release (↑/↓)	Specific NOS isoform(s) implicated (if known)	References
Human saphenous vein endothelial cells	CB1R	↓	iNOS	Stefano et al. (1998)
Microglial cells	CB1R	↓	iNOS	Waksman et al. (1999), Cabral et al. (2001)
C6 cells	CB1R	↓	iNOS	Esposito et al. (2006)
CGCs	CB1R	↓	nNOS	Hillard et al. (1999)
Neurons	CB1R and/or CB2R	↓	?	Molina-Holgado et al. (2002), Ribeiro et al. (2013)
Rat placenta	CB1R and CB2R	↓	?	Cella et al. (2008)
Rat medullary thick ascending limb suspensions	CB1R	↑	?	Silva et al. (2013)
Rat median eminence	CB1R	↑	?	Prevot et al. (1998)
Human monocytes	CB1R	↑	?	Stefano et al. (2000)
N18TG2 neuroblastoma cells	CB1R	↑	nNOS	Carney et al. (2009)
Gastric tissue	CB1R	↑	?	Rutkowska and Fereniec-Golebiewska (2009)
Kidney (mouse)	CB1R	↑	iNOS	Mukhopadhyay et al. (2010a)
Heart tissue (mouse)	CB1R	↑	?	Mukhopadhyay et al. (2010b)
Kidney (mouse)	CB2R	↓	iNOS	Mukhopadhyay et al. (2010c)
Macrophages	CB2R	↓	?	Ross et al. (2000)
Rat precerebellar neurons	CB2R	↓	iNOS	Oddi et al. (2012)
Rat heart	CB2R	↓	iNOS	Gonzalez et al. (2011)
Rat precerebellar neurons	CB2R	↑	nNOS	Oddi et al. (2012)
Neonatal cardiac cells	CB2R	↑	iNOS	Shmist et al. (2006)
Rat heart	CB2R	↑	eNOS	Gonzalez et al. (2011)
Isolated rat mesenteric arterial bed	TRPV1	↑	?	Poblete et al. (2005)
Rat placenta	TRPV1	↑	?	Cella et al. (2008)

The table outlines the reported effects of CB1R, CB2R, or TRPV1 activation upon NO^•^ production and/or release in the cell types/tissues listed. The involvement of the different ECS components (i.e. CB1R, CB2R, or TRPV1) is inferred from the use of selective agonists and/or through their genetic or pharmacological inhibition in the studies cited. Also included are details regarding the specific NOS isoform(s) involved if known. Arrows indicate increased (↑) or repressed (↓) NO^•^ production as evidenced/inferred by changes in NO^•^ levels, effects of NOS inhibition, altered NOS expression, and/or nitrosative stress.

### CB1R-mediated regulation of NO^•^ signalling

Several independent studies provide evidence that CB1R activation can act to suppress NO^•^ synthesis. For example, Molina-Holgado et al. (1997) demonstrated that LPS-induced release of NO^•^ in primary mouse astrocytes is attenuated by AEA and the synthetic cannabinoid agonist CP 55,940 in a CB1R-dependent manner. Notably, this response coincided with the ability of these cannabinoid ligands to negate LPS-induced iNOS expression (mRNA and protein). Moreover, NO^•^ production by iNOS in response to inflammatory stimuli has similarly been shown to be suppressed following CB1R activation in saphenous vein endothelial cells (Stefano et al., 1998), microglial cells (Waksman et al., 1999; Cabral et al., 2001), and neurons (Esposito et al., 2002; Molina-Holgado et al., 2002; Sheng et al., 2005; Fernandez-Lopez et al., 2006; Ribeiro et al., 2013). Notably, it has been suggested that in microglia, the ability of AEA to suppress iNOS activation may be mediated, at least in part, through CB1R-induced activation of MAPK phosphatase-1 (MKP-1), a proposed negative regulator of iNOS (Wang et al., 2009; Krishnan and Chatterjee, 2015). It is noteworthy that these suppressive effects of CB1R signalling upon NOS activity are not restricted to the iNOS isotype. For example, the CB1R agonists WIN 55,212-2, CP 55,940, and HU-210 have also been reported to inhibit KCl-induced activation of nNOS in cerebellar granule cells (CGCs) (Hillard et al., 1999). Importantly, this impaired activation of nNOS in response to CB1R stimulation in CGCs coincided with reduced influx of calcium through voltage-operated calcium channels following membrane depolarization (Hillard et al., 1999). Consistent with these findings, mice deficient for CB1R exhibit increased total NOS activity in the cerebral cortex (subventricular zone) compared to wild-type counterparts, concomitant with reduced neurogenesis in the denate gyrus and subventricular zone (Kim et al., 2006). Notably, this impaired neurogenesis observed in CB1R-deficient mice was reversed following administration of the nNOS-preferring inhibitor 7-nitroindazole. In addition, independent work by Nozaki and colleagues revealed that mechanical allodynia and neuronal activation of the trigeminal nucleus induced by the NO^•^ donor nitroglycerin were completely abolished in mice deficient for FAAH, the main enzyme responsible for degrading AEA (Kim et al., 2006). Notably, the nitroglycerin-induced effects were found to be restored in the FAAH knockout mice following inhibition of CB1R by SR141716 (Kim et al., 2006).

In contrast to the repressive effects of ECS activation upon NO^•^ formation, evidence exists to suggest that ECS stimulation may act to increase NO^•^ levels under certain conditions and/or in specific cell types. For example, AEA has been reported to stimulate NO^•^ generation and/or release in human monocytes (Stefano et al., 1996), rat median eminence fragments (Prevot et al., 1998), human saphenous vein segments (Stefano et al., 1998), and cultured human endothelial cells (Stefano et al., 1998; Fimiani et al., 1999; Mombouli and Vanhoutte, 1999). In the study by Prevot et al. (1998), increased NO^•^ production in rat median eminence fragments in response to AEA stimulation was found to be dependent upon CB1R activity. Moreover, 2-AG treatment has been shown to induce an immunosuppressive response in human monocytes and immunocytes from *Mytilus edulis* coinciding with a CB1R-mediated increase in NO^•^ release (Stefano et al. 2000). In a separate study by Carney et al. (2009), stimulation of CB1R by the cannabinoid receptor agonists CP 55,940 and WIN 55,212-2 elevated NO^•^ production via nNOS in N18TG2 neuroblastoma cells. CB1R-induced NO^•^ production has also been implicated in mediating the ability of ACEA to protect against aspirin-induced gastric ulceration in rats (Rutkowska and Fereniec-Golebiewska, 2009). Consistent with these findings, doxorubicin-induced oxidative/nitrosative stress has also been reported to be mitigated in the hearts of CB1R knockout mice (Mukhopadhyay et al., 2010c). Therefore, CB1R can act to positively or negatively modulate NO^•^ production depending on cell/tissue type and context.

### Regulation of nitrergic signalling by CB2R

Several studies have also implicated a role for CB2R in the modulation of NO^•^ production and/or release. For example, CB2R agonist treatment has been reported to attenuate cisplatin-induced iNOS expression and nitrosative stress in mouse kidneys (Mukhopadhyay et al., 2010a). In accord with this, inhibition of LPS-induced NO^•^ release in macrophages by WIN 55,212-2 was shown to be mediated through stimulation of CB2R (Ross et al., 2000). Interestingly, independent work by Oddi et al. (2012) revealed that daily treatment of rats with the CB2R agonist JWH-015 for one week markedly attenuated hemicerebellectomy induction of iNOS expression and associated oxidative/nitrosative stress, concomitant with improved neurological outcome measures. Intriguingly, Oddi et al. (2012) further demonstrated that JWH-015 treatment increased nNOS expression and activity in axotomized neurons, and the observed neuroprotection conveyed by CB2R activity was abrogated in response to pharmacological inhibition of nNOS.

Conversely, the naturally occurring cannabinoid compound delta-9-tetrahydrocannabinol (Δ^9^-THC) has been reported to increase NO^•^ production in neonatal cardiac cells through the induction of iNOS activity in a CB2R-dependent manner, thereby protecting cardiac cells from hypoxic damage (Shmist et al., 2006). Notably, the NOS inhibitor N_ω_-Nitro-L-arginine methyl ester (L-NAME) was found to block this Δ^9^-THC-induced cardioprotective action (Shmist et al., 2006). In accord with this, administration of the cannabinoid receptor agonist WIN 55,212-2 has also been shown to improve cardiac recovery following ischaemia/reperfusion (I/R) injury in the hearts from Zucker diabetic fatty rats by restoring coronary perfusion pressure and heart rate to pre-ischaemic levels (Gonzalez et al., 2011). Importantly, this cardioprotective action concurred with a reduction in cardiac iNOS expression whilst increasing eNOS expression in diabetic hearts subject to I/R injury. Moreover, the WIN 55,212-2-mediated cardiac recovery was completely blocked by the CB2R antagonist AM-630, thereby indicating a key cardioprotective role for this receptor (Gonzalez et al., 2011).

Although it remains unclear why CB1R/CB2R stimulation should convey such disparate actions upon nitrergic signalling (i.e. positive or negative regulation of NO^•^ production) in distinct cell/tissue types or in response to different stimuli, this may be due to the differential modulation of pathways that have been implicated in NO^•^ production. For example, CB1R activation has been shown to either promote the accumulation or impair production of intracellular cAMP in different cell types, which in turn may accentuate or relieve protein kinase A (PKA)-mediated inhibition of NOS-generating enzymes, respectively (Glass and Felder, 1997; Hampson and Grimaldi, 2001). Alternatively, it is possible that tissue-selective expression of specific NOS isoforms may also impact upon ECS modulated NO^•^ production, whereby the extent of coupling of ECS signalling to the activation of certain NOS isotypes may be more prominent in certain cell types.

### Alternative targets involved in the modulation of NO^•^ production

ECS ligands may also promote their biological actions by binding to alternative molecular targets, including the non-selective cation channel TRPV1 (Miyashita et al., 2012). For example, AEA has been reported to modulate NO^•^ levels in rat placenta by two independent mechanisms, either by decreasing NOS activity through stimulation of CB1R/CB2R, or alternatively, by upregulating NO^•^ formation via TRPV1 activation (Cella et al., 2008). In addition, both AEA and the TRPV1 agonist capsaicin have been shown to stimulate NO^•^ release in the isolated rat mesenteric arterial bed, with their stimulatory effects being attenuated following co-incubation with TRPV1 antagonists (Poblete et al., 2005). Moreover, a separate study by Luce et al. (2014) demonstrated the ability of AEA to decrease oxytocin and vasopressin secretion from neurohypophysis in adult rats. Notably, the inhibitory actions of AEA were found to be negated using the NOS inhibitor L-NAME, as well as CB2R and TRPV1 antagonists (Luce et al., 2014). These findings reveal a potential link between endocannabinoid, NO^•^, and oxytocin/vasopressin-induced signalling, which may serve to modulate homoeostatic, behavioural, and reproductive processes (Luce et al., 2014). Furthermore, it is possible that modulation of NO^•^ production and associated nitrergic signalling may be mediated through the reported ability of ECS ligands to activate PPAR isoforms (O'Sullivan, 2007; Prakash et al., 2015), although no direct evidence for this has yet been obtained. Alternatively, metabolites derived from endocannabinoids, such as prostaglandins, may also participate in the modulation of NO^•^ formation by the ECS (Urquhart et al., 2015). For example, the polyunsaturated omega-6 fatty acid arachidonic acid (C20:4n-6) produced following AEA degradation by FAAH serves as a substrate for cyclooxygenases, which in turn catalyze the formation of prostaglandins. Notably, evidence from several studies indicates that prostaglandins can stimulate or inhibit NOS activity, depending on cell type and context (Milano et al., 1995; Ribeiro et al., 2004; Cella et al., 2006). However, whether endogenous ECS ligands can modulate NOS activity via the action of prostaglandins and/or other derived metabolites remains unknown.

## Nitrergic modulation of ECS function

It is important to highlight that in addition to the growing body of evidence implicating a regulatory role for the ECS in modulating NO^•^ production and associated nitrergic signalling, there are also several studies suggesting that RNS may act to alter ECS function. For example, a study by Kokkola et al. (2005) using [^35^S]-GTPγS autoradiography and membrane binding assays revealed that treatment with the NO^•^ donor S-nitroglutathione (GSNO) led to the inhibition of CP 55,940 and WIN 55,212-2-induced CB1R signalling in the cerebral cortex, hippocampus, and the globus pallidus regions of rat brain. It is plausible that the inhibitory action of GSNO may be due to NO^•^-induced post-translation modifications of the CB1 receptor, a possibility that would require further analysis. In addition to the reported effects on CB1R function, work by Hervera et al. (2010) has also implicated nNOS-derived NO^•^ in the regulation of CB2R gene transcription during neuropathic pain, whereby sciatic nerve injury was shown to increase CB2R mRNA abundance in spinal cords of wild-type and iNOS-KO mice relative to naive (control) mice, but not in nNOS-deficient animals. In a separate study, rats administered with the NO^•^ donor nitroglycerin were found to exhibit increased heart tissue content of the endocannabinoid 2-AG (Wagner et al., 2006). Notably, this elevation in cardiac 2-AG abundance coincided with nitroglycerin-mediated protection against myocardial infarction, an effect shown to be dependent upon CB1R activity. Allied to this, administering 2-AG or its metabolically stable derivative noladinether before I/R mimicked the cardioprotective effects of nitroglycerin and similarly reduced infarct size (Wagner et al., 2006).

In addition, a study performed in isolated bovine brain microvessels, an *ex vivo* model of the blood–brain barrier, revealed that stimulation of CB1R enhances the activity of AMT, a selective AEA membrane transporter protein, by increasing NOS activity and NO^•^ production (Maccarrone et al., 2006). In the same study, AMT activity was shown to be reduced following CB2R stimulation, concomitant with suppressed NOS activity and NO^•^ release. Allied to this, immunoimaging revealed that different endothelial cells vary in the expression of CB1R and CB2R on their luminal and/or abluminal sides (Maccarrone et al., 2006). Such differential localization of cannabinoid receptors may facilitate coordinated AMT activity on the luminal and abluminal membranes, thereby promoting directional transport of AEA through the blood–brain barrier and other endothelial cells (Maccarrone et al., 2006). Collectively, these findings indicate that elevated levels of RNS may influence ECS function by altering the expression, activity, and/or localization of its key components.

## ECS**–**NO^•^ crosstalk in disease development and treatment

**Figure 3:**
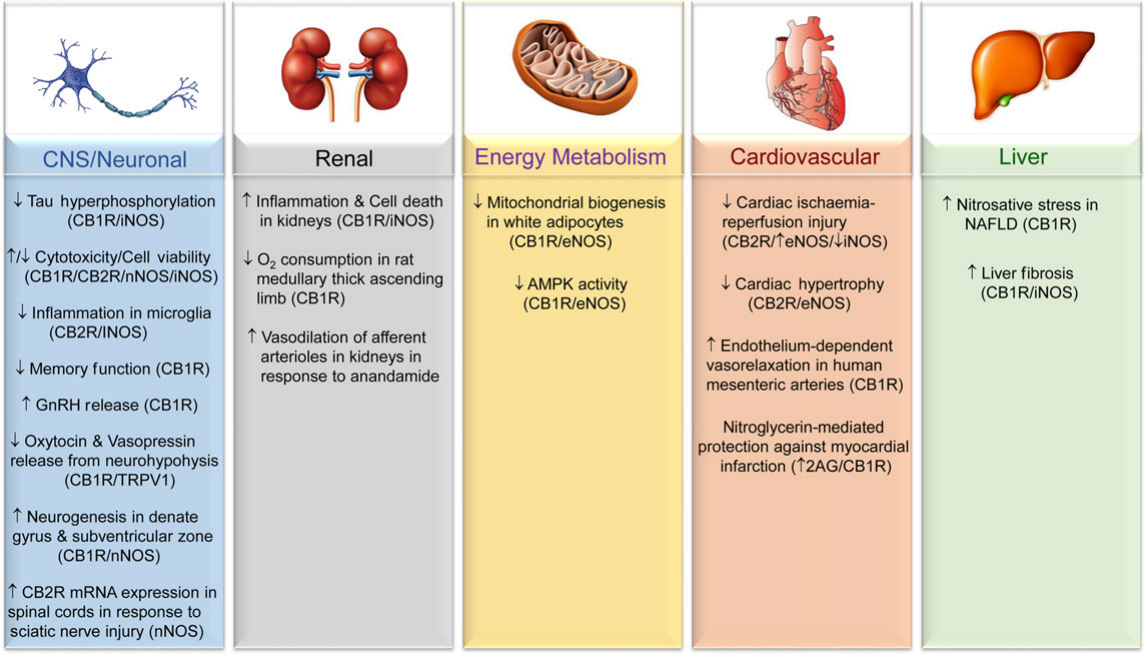
Involvement of the ECS**–**NO^•^ signalling axis in disease development and/or treatment. Schematic outlines the reported involvement of ECS**–**NO^•^ signalling in the tissues/processes presented. The participation of different ECS components (i.e. CB1R, CB2R, or TRPV1) is inferred from the use of selective agonists and/or through genetic or pharmacological inhibition, and is presented in brackets if known. Also included are details of the specific NOS isoform(s) involved in mediating the indicated response if determined. Arrows denote an inferred induction (↑) or repression/alleviation (↓) of the indicated process/pathology in response to activation of the specified ECS component.

Given the reported involvement of the ECS and NO^•^ signalling in the modulation of cellular function and the development of various physiopathological processes, it is likely that crosstalk between these signalling systems may contribute to nitrosative stress-related disease initiation and/or progression. These potential links are discussed in the following sections and summarized in Figure 3.

### Involvement of ECS**–**NO^•^ crosstalk in disorders of the central nervous system

Regarding pathologies of the central nervous system (CNS), ligand-induced activation of CB1R and/or CB2R has been reported to convey either cytotoxic or cytoprotective actions in neurons and astrocytes, depending on cell stimulus, through altered NO^•^ production (Molina-Holgado et al., 2002; Duncan and Heales, 2005; Esposito et al., 2006; Shmist et al., 2006; Carney et al., 2009; Oddi et al., 2012; Aguirre-Rueda et al., 2015). Notably, elevated levels of AEA in the brain in response to FAAH inhibition have been associated with enhanced neuronal survival and reduced neurodegeneration in a mouse model of traumatic brain injury, coinciding with a reduction in the number of iNOS-expressing (activated) microglia in the ipsilateral cortex (Tchantchou et al., 2014). Furthermore, Gomez-Galvez et al. (2016) demonstrated that stimulation of CB2R in mice administered with HU-308 led to reduced inflammation in the striatum of LPS-lesioned mice, concomitant with the attenuation of LPS-induced iNOS gene expression. Notably, these findings are in agreement with the reported ability of CB2R activation to inhibit NO^•^ production in RAW264.7 macrophages (Ross et al., 2000).

Evidence also exists showing that activation of CB1R in CA1 pyramidal neurons impairs dendritic integration of excitatory inputs and spatial memory formation through stimulation of hyperpolarization-activated cyclic nucleotide-gated channels that underlie the H-current (Maroso et al., 2016). In this case, the latter has been reported to function as a key modulator of dendritic excitability that relies upon NOS-driven NO^•^ formation (Maroso et al., 2016). Independent work by Prevot et al. (1998) also demonstrated that CB1R activation by AEA can stimulate NO^•^ release from endothelial cells of median eminence fragments in rats, thereby promoting enhanced secretion of gonadotropin-releasing hormone (GnRH), a neurohormone that plays a pivotal role in controlling reproductive function. Furthermore, work by Esposito et al. (2006) revealed that CB1R stimulation counters increased iNOS protein and NO^•^ production in β amyloid-stimulated rat C6 glioma cells. Notably, this CB1R-mediated response coincided with inhibition of NO^•^-dependent hyperphosphorylation of tau, a microtubule-associated protein implicated in neurofibrillary tangle formation and the development of Alzheimer's disease, in co-cultured PC12 neurons (Esposito et al., 2006). Collectively, these studies provide emerging evidence supporting an important role for the ECS**–**NO^•^ signalling axis in controlling the physiological actions and/or viability of key cellular components of the CNS (i.e. neurons, astrocytes, microglia, brain microvascular endothelial cells), thereby impacting on cognitive and neuroendocrine function, as well as implicating the ECS**–**NO^•^ pathway as a potential therapeutic target to alleviate neurodegeneration in response to brain injury and neurological conditions such as Parkinson's disease, vascular dementia, and Alzheimer's disease (Koppel et al., 2014; Aso et al., 2016; Austin and Katusic, 2016; Gomez-Galvez et al., 2016; Jayant and Sharma, 2016).

### Modulation of pathological changes in peripheral tissues and organs by ECS**–**NO^•^ signalling

ECS and NO^•^-dependent signalling has also been shown to modulate the physiology of various other organ systems, including the cardiovascular and renal systems and those implicated in the regulation of energy homoeostasis (Nisoli et al., 2004; Batkai et al., 2007a; Tedesco et al., 2008; Mukhopadhyay et al., 2010a; Gonzalez et al., 2011). For example, genetic deletion and pharmacological inhibition of CB1R has been reported to mitigate cisplatin-induced inflammation and cell death in mouse kidneys, concomitant with improvements in renal function (as evidenced by the attenuation of elevated creatinine and serum blood urea nitrogen levels) (Mukhopadhyay et al., 2010a). Notably, this reported ability of CB1R blockade to alleviate renal dysfunction was associated with the diminution of iNOS expression and nitrotyrosine levels in the kidney following cisplatin treatment (Mukhopadhyay et al., 2010a). In a separate study by Silva et al. (2013), CB1R stimulation was shown to reduce oxygen consumption, a key determinant of active sodium chloride reabsorption, in rat medullary thick ascending limb suspensions, concomitant with increased NO^•^ production. Furthermore, AEA has also been reported to induce vasodilatation of afferent arterioles of the kidney by increasing endothelial release of NO^•^ (Deutsch et al., 1997).

NO^•^-dependent signalling may also underlie, at least in part, some of the metabolic responses conveyed by altered ECS activity. For example, the CB1R agonist ACEA has been reported to reduce eNOS expression in white adipocytes, concomitant with a reduction in mitochondrial biogenesis (Tedesco et al., 2010). Consistent with this, genetic and pharmacological inhibition of CB1R has been associated with the induction of eNOS-dependent mitochondrial biogenesis in adipocytes (driven by increased eNOS mRNA and protein abundance), coinciding with enhanced activation of AMP-activated protein kinase, a key positive regulator of fatty acid oxidation, in cultured adipocytes and white adipose tissue of high fat-fed mice (Tedesco et al., 2008). Furthermore, it is possible that CB1R inhibition may further stimulate eNOS by enhancing the activity of protein kinase B (PKB, also known as Akt), which has been identified as a positive modulator of eNOS function by phosphorylating its Ser617 and Ser1177 residues (Dimmeler et al., 1999; Fulton et al., 1999; Michell et al., 2002). In accord with this, CB1R stimulation or blockade has been associated with increased or reduced PKB/Akt activity, respectively (Esposito et al., 2008; Eckardt et al., 2009; Lipina et al., 2016). However, such a link between CB1R-regulated PKB/Akt signalling and eNOS activity has not yet been established. Interestingly, AEA has been shown to increase insulin-stimulated glucose uptake in differentiated 3T3-L1 adipocytes through a CB1R-dependent mechanism involving NOS activity; however, the involvement of PKB/Akt was not determined in this study (Gasperi et al., 2007). Given the fact that NO^•^ has been implicated in the regulation of insulin sensitivity and insulin secretion, as well as other key metabolic processes including glucose utilization, lipogenesis, and inflammation (Henningsson et al., 2002; Nakata and Yada, 2003; Razny et al., 2011; Trevellin et al., 2014; Li et al., 2016), further work will required to determine the extent to which NO^•^-dependent signalling contributes towards ECS-induced regulation of such pathways that impact upon energy homoeostasis and metabolism (Cinar et al., 2014; Gonzalez-Mariscal et al., 2016; Lipina et al., 2016).

It is possible that ECS**–**NO^•^-regulated metabolic responses may also contribute to the development and/or attenuation of related pathological conditions including non-alcoholic fatty liver disease (NAFLD) and cardiac dysfunction (Gonzalez et al., 2011; Jorgacevic et al., 2015; Nozaki et al., 2015). For example, CB1R inhibition by SR141716 has been reported to improve hepatic oxidative/nitrosative stress in mice with NAFLD induced by high fat feeding (Jorgacevic et al., 2015). The work of Gonzalez et al. (2011) also revealed that CB2R activation serves to mitigate cardiac I/R injury in Zucker diabetic rats by restoring cardiac iNOS/eNOS equilibrium (by decreasing or increasing cardiac iNOS and eNOS expression, respectively). Cardioprotection has also been reported to be conveyed by the NO^•^ donor nitroglycerin, as evidenced by its ability to mitigate myocardial infarction in rats through stimulation of CB1R activity in response to increased 2-AG levels in the heart (Wagner et al., 2006). Therefore, reciprocal regulation of ECS and NO^•^-induced signalling may play a pivotal role in controlling cardiomyocyte function and/or viability. In addition to these cardioprotective actions, ligand-induced activation of CB1R and CB2R has also been documented to attenuate hypertrophy of neonatal rat cardiomyocytes by a mechanism involving eNOS activity (Lu et al., 2014). Recent work by Stanley et al. (2016) also demonstrated the ability of AEA to promote endothelium-dependent vasorelaxation in human mesenteric arteries via a CB1R and NO^•^-dependent pathway. Therefore, these studies highlight the different ways that ECS**–**NO^•^ signalling axis can impact upon cardiovascular function.

Importantly, however, the extent to which altered ECS function, such as that observed in response to obesity or ageing (Batkai et al., 2007b; Lipina et al., 2016), contributes to pathology-driven changes in NO^•^ signalling, particularly *in vivo*, remains poorly understood. Further work utilizing relevant mouse models of NOS deficiency, for example, may provide a better understanding of the role that NO^•^-dependent signalling plays in mediating ECS-induced changes in the function of the CNS and extraneural tissue systems. To this end, combined therapeutic targeting of ECS components and NOS isoforms may provide a more effective strategy at preventing and/or treating certain pathologies. This is exemplified through recent work by Cinar et al. (2016) reporting the ability of a hybrid inhibitor of peripheral CB1R and iNOS to mitigate liver fibrosis induced by CCl_4_ or bile duct ligation in mice. Notably, Cinar et al. (2016) demonstrated that the hybrid CB1R/iNOS antagonist surpassed the antifibrotic efficacy of the CB1R antagonist SR141716 or the iNOS inhibitor 1400W. Subsequent work utilizing a similar approach by dual targeting of the ECS and NO^•^ systems may reveal further alterations to physiological and/or pathological responses in the liver (e.g. steatosis, inflammation) and other peripheral tissues.

Little is known of how altered ECS function may impact upon protein modifications mediated by NO^•^ and other RNS leading to protein nitration, nitrosation, or S-nitrosylation, which have been implicated in disease pathology (Beckman and Koppenol, 1996; Levonen et al., 2001; Soetkamp et al., 2014; Pereira et al., 2015; Seneviratne et al., 2016). To this end, further detailed analysis using mass spectrometry and/or other techniques will be required to establish how the ‘nitro-proteome’ is influenced by changes in ECS activity, which may also involve altered enzymatic activity that functions to degrade specific nitro group modifications including the removal of S-nitrothiols by S-nitroglutathione reductase. Importantly, such work may identify novel targets of ECS**–**NO^•^ signalling that contribute to disease pathogenesis.

## Funding

Research in the authors' laboratory is supported by the Biotechnology and Biological Sciences Research Council and Diabetes UK.

## Conclusion

Collectively, the evidence presented in this review indicates that ECS activation or inhibition can convey detrimental and/or beneficial biological actions by altering cellular NO^•^ production and/or release, depending on cell type and/or stimulus. The studies highlighted in this review demonstrate that ECS activity can modulate NO^•^ production and associated downstream processes in a number of different ways (Figures 2B and 3). Moreover, we draw attention to emerging evidence for the reciprocal modulation of ECS function by RNS. Crucially, given the importance of nitrergic signalling and the ECS in the development of numerous pathologies (Figure 3), these findings identify ECS components as potential therapeutic targets for the treatment of nitrosative stress-related neurological, cardiovascular, and metabolic disorders. Consequently, it will be of great interest to define the molecular targets of ECS**–**NO^•^ signalling in distinct tissues under various pathological states and to explore how ECS-induced nitrergic signalling and/or nitrosative stress may contribute to alterations in the function(s) of proteins implicated in disease initiation and progression.
